# Immune Mediators Important for a Protective Secondary Response to *Babesia microti*

**DOI:** 10.3390/pathogens13020123

**Published:** 2024-01-28

**Authors:** Joseph Conti, Thomas Gagliardi, Paul M. Arnaboldi, Synthia J. Hale, Sini Skariah, Ali A. Sultan, Dana G. Mordue

**Affiliations:** 1 Department of Pathology, Microbiology and Immunology, New York Medical College, Valhalla, NY 10595, USA; jconti@student.nymc.edu (J.C.); tgagliar@student.nymc.edu (T.G.); paul_arnaboldi@nymc.edu (P.M.A.); jeanettejhale@gmail.com (S.J.H.); 2 Department of Microbiology and Immunology, Weill Cornell Medicine-Qatar, Doha 2713, Qatar; siniskariah@gmail.com (S.S.); als2026@qatar-med.cornell.edu (A.A.S.)

**Keywords:** *Babesia*, *Babesia microti*, adaptive immunity, babesiosis, erythrocytes

## Abstract

*Babesia microti* (*B. microti*) is a tick-transmitted protozoan parasite that invades red blood cells. It is the primary cause of human babesiosis in the US. The severity of babesiosis caused by *B. microti* infection can range from asymptomatic to fatal. Risk factors for severe disease include general immune suppression, advanced age (>50) and lack of a spleen. However, severe disease can occur in the absence of any known risk factors. The degree to which tick-transmitted *B. microti* infection confers protection from subsequent exposure is largely unexplored. This is an important question as both the prevalence and geographic range of tick-transmitted *B. microti* infection continues to increase and individuals in endemic regions may have multiple exposures over their lifetime. In the current study we used a mouse model to evaluate the degree to which primary infection with *B. microti* protected against secondary challenge with the same parasite strain. We show that CD4 T cells, and to a lesser extent B cells, contribute to protection. However, mice exhibited significant protection from secondary parasite challenge even in the absence of either CD4 T cells or B cells. The protection mediated by CD4 T cells did not depend on their production of IFN-γ as mice with a targeted gene deletion for the IFN-γ receptor remained fully protected against secondary challenge. Other factors including inducible nitric oxide synthase (iNOS) and the adaptor protein MyD88, important for toll-like receptors, IL-18 and IL-1 signaling, were not important for protection against primary or secondary challenge with *B. microti.* Thus, our study shows that resolution of primary infection with *B. microti* results in robust protection against secondary challenge with parasites, at least in the short term. Further studies are needed to evaluate the length of protection and the degree to which protection is impacted by parasite heterogeneity. Although we show an important role for CD4 T cells in protection against secondary challenge, our results suggest that no single aspect of the immune system is solely responsible for adequate protection against secondary challenge with *B. microti*.

## 1. Introduction

The genus *Babesia* is part of the Babesiidae family and Piroplasmida order [1,2]. Members of the Piroplasmida are common infectious agents for humans as well as domestic and wild animals. The species implicated in human disease can be classified into distinct clades [1,3,4,5]. There is evidence that severity of infection, immunity and drug efficacy may differ depending on the clade of *Babesia* as well as the species [2,6,7,8]. *B. microti* is a member of the *Babesia microti*-like clade that is also sometimes referred to as small *Babesia.* Small *Babesia* in North America are primarily transmitted by *Ixodes scapularis* ticks. In contrast, in Europe small *Babesia* are primarily transmitted by *Ixodes ricinus* ticks [9]. The primary agent of human babesiosis in North America is *B. microti* while the most common agent in Europe is *B. divergens* [9].

*B. microti* invades red blood cells and forms a transient parasitophorous vacuole (PV) from which it immediately escapes to reside and replicate by binary fission in the erythrocyte cytosol. Unlike *Plasmodium* species, *B. microti* is not known to have an extra-erythrocytic stage and appears to remain in circulation, within erythrocytes, for the duration of the infection. Disease severity due to *B. microti* ranges from asymptomatic to severe infections which require hospitalization and can result in death [8,10,11]. Known factors that increase susceptibility to severe disease are an age greater than 50, general immune suppression, and lack of a spleen [8,12]. Treatment failures, including development of drug-resistant parasites and prolonged parasitemia, are most common in immune suppressed individuals [12,13,14,15]. The immunopathology of babesiosis remains poorly defined, and as such, other risk factors and determinants for severe disease remain unknown.

In a prior study, we evaluated *B. microti* infection in C3H, BALB/c and C57BL6 mice to determine if differences in the genetic background of the mice impacted peak parasitemia and resolution of infection as defined by clearance of parasitemia [16]. Peak parasitemia was lowest in C57BL6 mice and highest in C3H mice (range of 35–60% parasitemia depending on mouse strain) and occurred 7–10 days post-infection. However, all three mouse strains rapidly resolved parasitemia over a similar time course resulting in parasite clearance to undetectable levels by day 14–15 post-infection in immunologically intact animals, as determined by microscopic evaluation of blood smears. We used C57BL6 mice with targeted gene deletions to evaluate the importance of CD4 T cells, B cells, IFN-γ receptor (IFN-γ receptor KO), inducible nitric oxide synthase (iNOS) and pattern recognition receptor signaling (myeloid differentiation primary response 88 [MyD88]) KO mice), in the regulation of peak parasitemia and parasite clearance (resolution of infection). Our data supported previous findings that CD4 T cells are important for resolution of infection as CD4−/− mice did not clear the parasite and developed a persistent parasitemia. The absence of CD4 T cells did not impact levels of peak parasitemia. Mice deficient in the IFN-γ receptor or mature B cells exhibited no change in peak parasitemia and a short delay in parasite clearance compared to WT immunologically intact mice. MyD88 and iNOS knockout mice displayed no difference in peak parasitemia or clearance of the infection.

Limited studies in humans suggest that resolution of human *B. microti* infection may not result in protective immunity against a second infection [17]. In the present study, we evaluated if mice that recently cleared a primary infection with *B. microti* were protected against a second infection with the same species. Using gene disrupted mice, we also tested the extent to which specific elements of the adaptive immune response, including CD4 T cells, B cells and IFN-γ, contribute to protection against secondary *B. microti* challenge.

## 2. Materials and Methods

### 2.1. Mice

Mouse strains used for this study were purchased from Jackson Laboratory (Bar Harbor, ME, USA). C57BL6 mice (Jackson Laboratory strain number 000664) without any targeted gene deletions are referred to as wild type (WT) mice throughout the text. All mice with targeted gene deletions are in the C57BL6 genetic background. Mice deficient in CD4 T cells have a targeted gene deletion for the cell surface marker CD4 (B6.129S2-*Cd4^tm1Mak^*/J Jackson Laboratory strain number 002663). MuMT mice have a targeted gene deletion that disrupts one of the membrane exons needed for expression of the IgM (mu) heavy chain in B cells. These mice lack IgM and most mature B cells, although a few B cells may be produced using a heavy chain other than IgM (Jackson Laboratory strain number 002288; B6.129S2-*Ighmtm1Cgn*/J). Mice deficient in the IFN-γ receptor have a targeted deletion of exon 5 for the IFN-γ receptor gene resulting in a loss of expression of the receptor for IFN-γ (Jackson Laboratory strain number 003288; B6.129S7-*Ifngr1*^tm1Agt^/J). Mice deficient in inducible nitric oxide synthase (iNOS) have a targeted gene deletion that replaces the calmodulin binding domain of iNOS with a neomycin resistance gene resulting in a loss of iNOS expression (Jackson Laboratory strain number 002596; B6.129P2-*Nos2*^tmaLau^/J). Mice deficient in the myeloid differentiation primary response 88 (MyD88) gene have a targeted deletion of exon 3 of the MyD88 gene resulting in the loss of expression of the MyD88 protein (Jackson Laboratory strain number 00908; B6.129P2 (SJL)-*Myd88*^tm.1.1Defr^/J). All animal studies were approved by the New York Medical College Institutional Animal Care and Use Committee (IACUC). The studies were approved in the NYMC IACUC protocol number 13958. All mice cages for an experiment were housed in the NYMC animal facility in the same room in the same rack and rack shelf to minimize potential environmental confounding factors. Mice were housed in groups of 4–5 per cage. The facility is maintained specifically pathogen free and screened quarterly using separate prevalent and comprehensive pathogen panels.

### 2.2. Parasite

The *B. microti* Gray strain, originally isolated from a human infection on Nantucket Island, Massachusetts in 1970, was obtained from the American Type Culture Collection (ATCC 30221) and was used for all of the studies [18]. Parasites were maintained by serial passage in C57BL6 WT mice after being rapidly thawed from storage in liquid nitrogen and injected intraperitoneally (IP) into two mice to allow parasites to recover from cryopreservation and replicate to sufficient numbers for serial passage in mice. *B. microti* currently can not be propagated long-term in vitro in red blood cell culture but must be propagated in mice or Golden Hamsters. Parasites were passaged for two rounds in mice before being used for challenge.

### 2.3. Primary and Secondary B. microti Challenge

The goal for each experiment was to assess the magnitude and duration of parasitemia in WT and KO mice after secondary challenge with *B. microti*. For each secondary challenge experiment, a group of naïve WT mice was given a primary challenge with *B. microti* using the same parasite inoculum as an internal control within each experiment for parasite infection and to compare parasitemia during primary versus secondary challenge within an experiment.

Power analysis determined that 4 mice per group was sufficient to achieve statistically meaningful data with a 2-fold effect size using an alpha of 0.05, 95% confidence and a power of 0.80. We assessed parasitemia longitudinally using 4–5 mice per group for each experiment. An n of 50 mice was used in total for the current study. All mice in each experimental group were included in the analyses with no exclusions. All mice received the same challenge with *B. microti* (same treatment) and differed only in whether the mice received a primary or secondary challenge and whether the mice were WT or KO mice.

**Figure 1:**
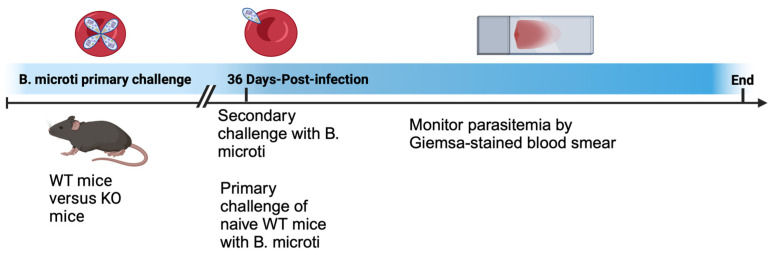
Schematic of primary and secondary challenges with *B. microti*.

For primary and secondary challenges with parasites, 4–5 mice per group were administered an IP injection of 100 μL pooled blood collected from infected mice 5 days post-infection when parasitemia was ~30%. Mice were challenged with ~1 × 10^7^ infected red blood cells. Secondary challenge with *B. microti* was performed 36 days post primary challenge unless otherwise stated. Parasitemia was assessed longitudinally during infection by collecting blood by tail snip of mice to create a thin film blood smear on a microscope slide. Blood smears were stained with Giemsa (Richard-Allan Scientific, Kalamazoo, MI, USA), and evaluated for parasites using brightfield microscopy imaging. The only outcome measured in the experiments was parasitemia. No mice were excluded from the analysis. A schematic of the experimental design is shown below in Figure 1 and was designed using BioRender (BioRender.com).

### 2.4. Hematology and Analysis of Parasitemia

The experimental unit used for each experiment was parasitemia in each individual mouse. Parasitemia was evaluated for each mouse by counting the number of parasites per 100 red blood cells in 3 different areas of the blood smear. Blood smears were evaluated using a Nikon Eclipse TiE inverted microscope with 100× oil objective and bright field illumination (Nikon Instruments Inc., Melville, NY, USA). Parasite counts per each mouse were conducted blind. In addition, assessment of parasitemia following secondary challenge was performed by individuals in the laboratory who were not involved in the study design or infection procedures.

The mean, standard deviation and standard error from mouse groups was determined for each time point. An unpaired Student’s *t*-test was used to compare the means of two groups of mice per time point (WT versus a single KO mouse strain) using GraphPad Prism 9. The data was assumed to be continuous, normally distributed with a homogeneity of variance. The assumption of normal distribution was based on the data in the current paper and similar primary and secondary challenge studies with an additional n = 50 WT C57BL6 mice and KO mice [16] not included in the current paper. Furthermore, we have analyzed *B. microti* parasitemia longitudinally during primary infection in additional published and unpublished studies that all showed a normal distribution of parasitemia in inbred mouse strains. The data in the paper are shown for each individual mouse rather than simply the mean and standard deviation for each group to aid in assessment of the variability between mice.

## 3. Results

In a previous study, we examined primary infection with *B. microti*, including the contribution of different cell types and immune factors in the regulation of peak parasitemia and resolution of infection using mice with targeted gene deletions [16]. In the current study, we extend these findings by evaluating whether previously infected mice are protected from secondary challenge with the parasite. A diagram of the experimental mouse model is shown in the methods section in Figure 1.

**Figure 2:**
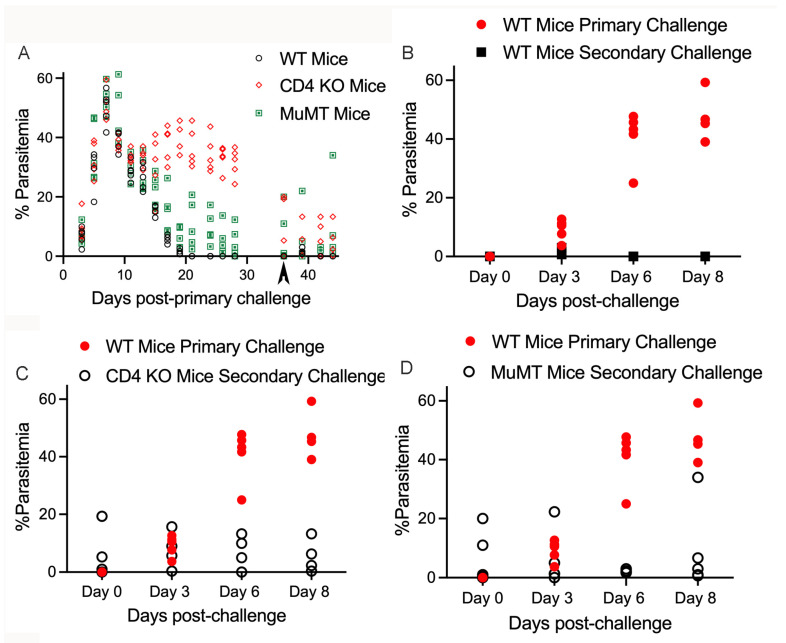
Evaluation of parasitemia in WT, CD4−/− (CD4 KO)and MuMT mice during primary and secondary challenges (day 36 pi) with *B. microti*. (**A**) Parasitemia in response to primary challenge and secondary challenge with *B. microti* on day 36 pi. (**B**–**D**) Parasitemia following primary challenge of naïve WT mice compared to secondary challenge of WT, CD4−/− and MuMT mice, respectively. Parasitemia levels are shown for individual mice and are the average of 3 counts of 100 red blood cells in a Giemsa-stained blood smear using a 100× oil objective and brightfield illumination. WT mice administered a primary challenge with *B. microti* are represented by a black circle with white fill in panel (**A**) and red filled circles in panels (**B**–**D**). WT mice upon secondary challenge are represented by a black square. MuMT mice are represented by a green square or black circle with white fill depending on the figure. CD4−/− mice are represented by a red diamond or black circle with white fill depending on the figure.

First, we tested the hypothesis that a primary infection with *B. microti* would provide protection against a secondary challenge with the same parasite isolate. Mice were given a primary challenge with *B. microti* parasites followed by a secondary challenge on day 36 post-primary challenge. As shown in Figure 2A, parasitemia levels in WT mice reached approximately 40% within the first 10 days of primary infection and then rapidly declined. Parasites were no longer detectable in blood smears by day 20 post-infection. Sixteen days later (day 36) the mice received secondary challenge.

In order to directly compare parasitemia between primary and secondary challenges, a group of naïve WT mice were given a primary challenge with *B. microti* using the same parasites. This also provided a control that the parasites were infectious at a normal level. Secondary parasite challenge was assessed on day 0, 3, 6 and 8 (36, 39, 42 and 44 days after primary challenge, respectively). Primary challenge of naïve WT mice resulted in parasitemia levels of approximately 40% (Figure 2B). In contrast, WT mice were protected against a secondary challenge. After secondary challenge, parasitemia remained below 1% and parasites were no longer detectable by day 6 or 8 post-secondary challenge (Figure 2B). These results indicate that mice that previously cleared a *B. microti* infection rapidly clear parasites upon second exposure and are protected against a secondary challenge with the same parasite strain.

Having established that primary infection of WT mice resulted in protection of mice from secondary challenge with *B. microti*, we examined the importance of CD4 and B cells in this protection using CD4−/− mice and MuMT mice, respectively. CD4−/− and MuMT mice exhibited parasitemia levels that peaked at approximately 45% parasitemia on day 8 post-primary challenge with parasites (Figure 2A). However, unlike WT mice, CD4−/− mice were impaired in their ability to resolve parasitemia and uniformly maintained parasitemia levels above 20% for CD4−/− mice. In contrast to CD4−/− mice, only 50% of the MuMT mice still had detectable parasitemia by 36 days post-primary challenge (Figure 2A). While CD4−/− mice did not clear infection, there was still a significant reduction of parasitemia observed during the primary response in naïve animals, indicating that CD4−/− mice were able to partially control the level of parasites over time. MuMT mice were more effective than CD4−/− mice in their ability to resolve parasitemia but less effective than WT mice. Thus, it is clear that CD4 T cells contribute to resolution of *B. microti* primary infection in the C57BL6 mouse background. The contribution of B cells to resolution of infection was less pronounced but still evident.

Upon secondary challenge, CD4−/− mice demonstrated significantly reduced parasitemia (less than 20%) compared to naïve WT mice (43–60% parasitemia), indicating that some level of protection developed in these animals (Figure 2C). MuMT mice developed a low level of parasitemia (<5%) with the exception of one MuMT mouse that reached parasitemia levels similar to naïve WT mice (Figure 2D). In summary, our results show that both CD4 T cells and B cells contribute to protection of mice in response to both primary and secondary parasite challenges. However, neither CD4 T cells or B cells appear to be absolutely required for protection against secondary challenge since both CD4−/− and MuMT mice had reduced parasite levels during secondary infection compared to primary infection despite their inability to clear the primary infection.

**Figure 3:**
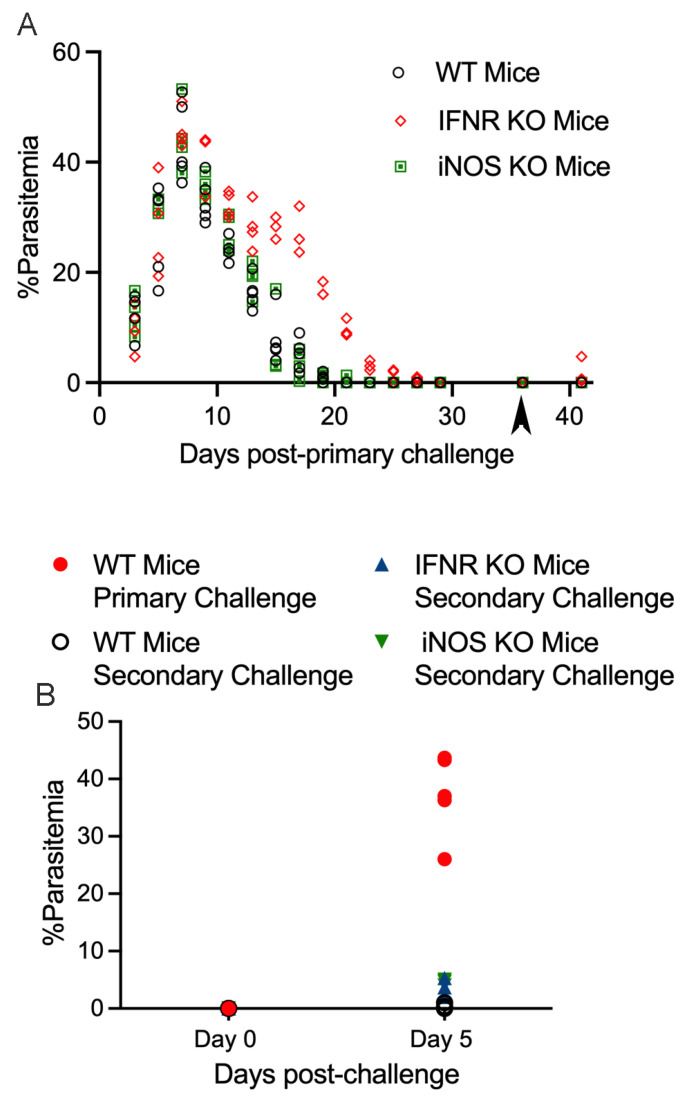
Evaluation of parasitemia in WT, IFN-γR−/−, and iNOS−/− mice during primary and secondary challenge (day 36 pi) with *B. microti*. (**A**) Parasitemia in response to primary infection with *B. microti* and secondary challenge with *B. microti* on day 36 pi. (**B**) Parasitemia following primary challenge of naïve WT mice compared to secondary challenge of WT, IFN-γR−/−, and iNOS−/− mice. In (**B**) WT mice are represented by a black circle with white fill. IFN-γR−/−, mice are represented by a red diamond or blue triangle depending on the figure. iNOS−/− mice are represented by a green square or green upside-down triangle depending on the figure. Parasitemia levels for each mouse are the average of 3 counts of 100 red blood cells in a Giemsa-stained blood smear using a 100× oil objective and brightfield illumination.

To evaluate the extent to which the protection mediated by CD4 T cells in primary and secondary challenges with *B. microti* is mediated by their production of IFN-γ, we used mice with a targeted deletion of the IFN-γR. WT or IFN-γR−/− mice were given a primary challenge with *B. microti* parasites followed by a secondary challenge on day 36 post-infection. Figure 3A,B shows parasitemia in WT mice in response to primary and secondary challenges with *B. microti*. The absence of IFN-γR resulted in only a 3-day delay in parasite clearance during primary infection and did not impact peak parasitemia levels. IFN-γR−/− mice were as resistant as WT mice to secondary challenge with *B. microti* (Figure 3A,B). This suggests that CD4 T cells are either not mediating protection against secondary challenge through the production of IFN-γ or that other redundant factors are capable of compensating for the lack of IFN-γ signaling in these mice. We also evaluated the importance of phagocyte production of nitric oxide using mice with a targeted deletion in inducible nitric oxide synthase (iNOS−/− mice). As shown in Figure 3, parasitemia levels following primary and secondary challenge of iNOS−/− mice were similar to WT mice. These results suggest that neither IFN-γR or iNOS plays a critical role in resistance of mice to secondary challenge with *B. microti*.

**Figure 4:**
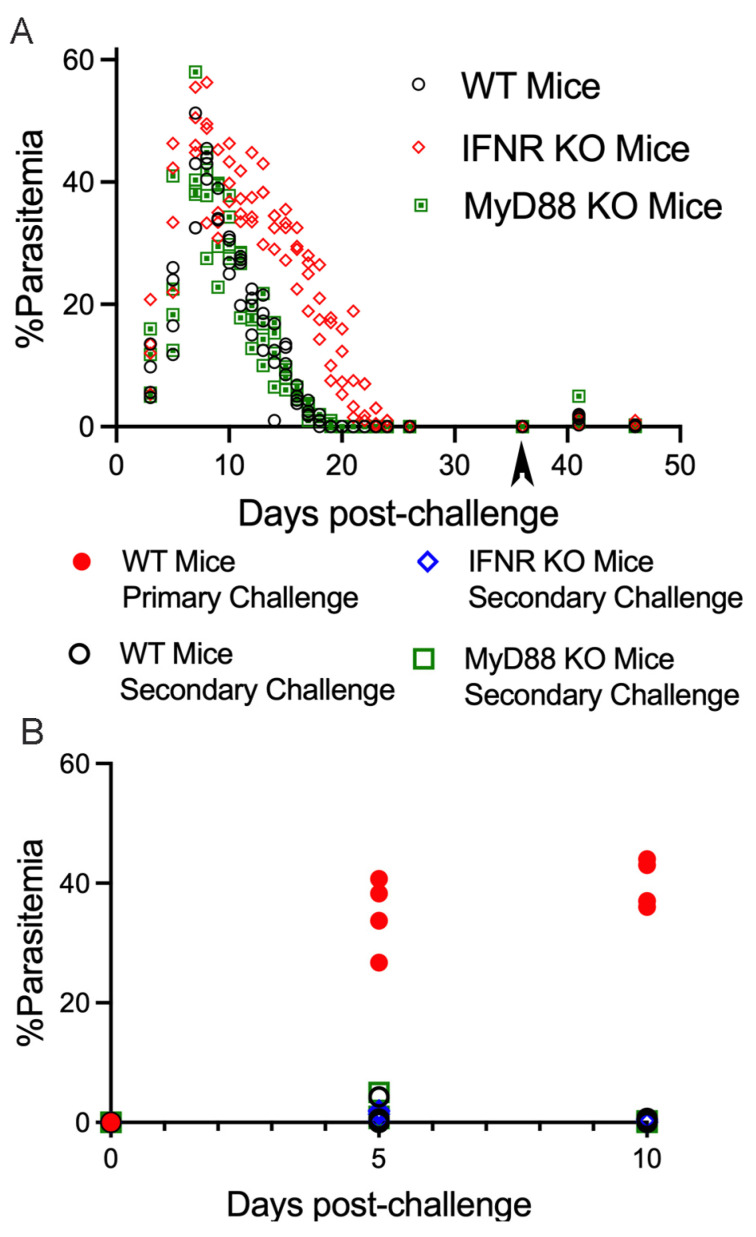
Evaluation of parasitemia in WT, IFN-γR−/−, and MyD88−/− mice primary and secondary challenges (day 36 pi) with *B. microti*. (**A**) Parasitemia in response to primary infection with *B. microti* and secondary challenge with *B. microti* on day 36 pi. WT mice are represented by a black circle with white fill. IFN-γR−/−, mice are represented by a red diamond. MyD88−/− mice are represented by a green square. (**B**) Parasitemia following primary challenge of naïve WT mice compared to secondary challenge of WT, IFN-γR−/−, and MyD88−/− mice. Parasitemia levels are shown for individual mice and are the average of 3 counts of 100 red blood cells in a Giemsa-stained blood smear using a 100× oil objective and brightfield illumination. Parasitemia in naïve WT mice administered a primary challenge with *B. microti* is shown as a red circle with red fill in (**B**). Parasitemia in response to secondary challenge of WT mice is represented by a black circle with white fill in (**A**,**B**). IFN-γR−/−, mice are represented by a blue diamond in (**B**). MyD88−/− mice are represented by a green square. Parasitemia values for individual mice are shown on day 0, 5 and 10 in naïve mice and for WT, IFN-γR−/− and MyD88−/− post-secondary challenge. There was a single MyD88−/− mouse that developed parasitemia on day 5 pi above 1%.

We next evaluated the role of TLRs and IL-1 and IL-18 signaling in protection against secondary challenge with *B. microti* using mice with a targeted gene deletion for myeloid differentiation primary response 88 gene (MyD88−/− mice). MyD88 is the main adaptor molecule for the majority of signaling through TLRs, and IL-1R and IL-18 family receptors [19]. We also repeated our analysis of the importance of IFN-γR in primary and secondary challenge with *B. microti* in the experiment with MyD88−/− mice. As shown in Figure 4, the absence of MyD88 did not impact parasitemia during primary infection with *B. microti*. MyD88 also was not required for protection against a secondary challenge with *B. microti* (Figure 4). Consistent with the experiment shown in Figure 3, IFN-γR−/− mice had a slight delay in parasite clearance during primary infection with *B. microti* and no impairment in protection against secondary challenge.

## 4. Discussion

There is little available information as to whether individuals previously infected with *B. microti* are protected against subsequent tick-transmitted babesiosis. However, a previous case report described an immune competent 62-year-old that suffered two separate episodes of *B. microti* babesiosis 3 years apart. The individual showed typical symptoms of babesiosis both times and evidence suggested reinfection with *B. microti* rather than reactivation of parasitemia from a single infection 3 years earlier [17]. In the current study, we evaluated whether mice recently infected with *B. microti* were resistant to a secondary parasite challenge. We also sought to determine which immune cell types and factors were important for protection against secondary challenge. Our study indicates that C57BL6 mice previously infected with *B. microti* were protected against a secondary challenge and mice either did not develop detectable parasitemia or developed a transient parasitemia below 1% after secondary challenge. The current study, in combination with our previous study of primary *B. microti* infection [16], indicates that CD4 T cells, and to a lesser extent B cells, contribute to resolution of primary infection as determined by clearance of parasitemia and protection against secondary challenge. However, even in the absence of CD4 T cells or B cells, mice were largely protected against secondary challenge with *B. microti*. This suggests that protection against *B. microti* is multifactorial and that no single aspect of the host immune response is solely responsible for protection.

Despite an ability to diminish but not clear primary infection with *B. microti* 36 days after the initial challenge, CD4−/− mice did not respond to secondary challenge with the high level of parasitemia observed in primary infection of naïve mice, indicating that they had developed at least some level of immunity capable of limiting the infection. This protection could be mediated by IgM antibodies, which are plentiful in CD4−/− mice (as they generally lack the ability to class switch antibodies). This possibility is consistent with our observation that at least some MuMT mice did develop parasitemia following secondary parasite challenge. Combined, these data suggest that antibodies along with CD4 T cells may contribute to the control of both primary *B. microti* infection and protection against secondary parasite challenge. An important function of CD4 T cells is production of IFN-γ that serves to activate macrophages and other cell types and initiate signaling for the transcription of IFN-stimulated genes, many of which have antimicrobial functions. In our current study, IFN-γR deficiency resulted in a modest delay in parasite clearance during primary infection but not in response to secondary challenge with parasites. This indicates that other factors important for resistance to secondary parasite challenge provide protection in the absence of IFN-γR. However, our results using iNOS−/− mice indicate that iNOS is not critical for resistance to primary or secondary challenge with *B. microti*. The fact that MyD88−/− mice showed no increase in susceptibility to either primary or secondary challenge with *B. microti* suggests that signaling through toll-like receptors or IL-1 and IL-18 receptors also do not play a critical role in resistance to *B. microti* in our model.

Both innate and adaptive immunity have been implicated in control of *B. microti* primary infection in mice [20,21,22,23,24,25]. Administration of BCG (Bacillus Calmette-Guerin) has been shown to protect mice from infection with *B. microti, B. rodhaini* as well as *Plasmodium berghei* and *Plasmodium vinckei* [20]. BCG protected mice from *B. microti* and *Plasmodium* species through the production of a non-antibody soluble mediator that killed parasites within infected red blood cells. Severe combined immunodeficient (SCID) mice have a genetic mutation that results in impaired VDJ rearrangement and have a functional defect in both mature B cells and T cells. SCID mice and nude mice deficient in T cells have also been shown to develop a persistent parasitemia in response to *B. microti* infection [21,24]. In general, this shows an important role for the adaptive immune response in resolution of *B. microti* infection as defined by clearance of parasites. However, the ability of SCID mice to control parasitemia also depends on the genetic background of the mice, suggesting that in some genetic backgrounds innate immunity may be of critical importance along with CD4 T cells [24]. A study in BALB/c mice showed that CD4 T cells, but not CD8 T cells or B cells, were important for resolution of *B. microti* primary infection with the KR-1 parasite strain [26]. Cell-mediated immunity did not require IFN-γ as IFN-γ−/− mice were able to clear infection although they did develop higher parasitemia than WT mice. Studies to date generally support an important role for CD4 T cells in control of parasitemia and/or clearance of parasites and resolution of infection. However, the importance of IFN-γ and B cells is highly variable between published studies.

Studies to date indicate that host and parasite genotype might play a significant role in the severity as well as resolution of *Babesia* spp. infections, and that the *Babesia* strain or clade has a significant impact on resistance/susceptibility and the immune cells and factors that contribute to the outcome of infection. Host genotype is clearly an important determinant of disease severity in response to infection with the WA1 *Babesia* strain that is related to the *B. duncani*-like parasites [27,28]. C57BL6 and C57BL10 mice have been shown to be highly resistant with an LD_50_ of 10^8^. BALBC/cJ mice were of intermediate susceptibility with an LD_50_ of 2 × 10^7^. In contrast, C3H mice were highly susceptible with an LD_50_ of 6 × 10^4^. The importance of the SCID mutation to resistance to infection with the *Babesia* WA1 strain is also mouse genotype dependent. The SCID mutation in the C3H mouse background had little impact on the susceptibility of C3H mice to WA1 infection as both WT and SCID mice were susceptible [24]. The SCID mutation in the C57BL6 mouse background did not impair the resistance of C57BL6 mice to WA1 infection, indicating that B and T cells were not required for protection against the WA1 parasite strain in C57BL6 mice. In contrast, the SCID mutation in the BALB/c background dramatically impaired the ability of the moderately susceptible BALB/c mouse strain to control infection. These studies suggest that host genotype may play a critical role in the requirement of innate versus adaptive immunity in the control of *B. duncani*-like parasites as well as in the susceptibility and resistance to the parasite.

Studies also suggest that host genotype impacts *B. microti* infection but perhaps not to the same degree as the WA1 parasite strain. Our previous study of primary infection with *B. microti* showed that host genotype impacted peak parasitemia levels in C3H, BALB/c and C57BL6 mice, but all three mouse strains were able to resolve infection over a similar longitudinal time frame [16]. An earlier study that examined the impact of mouse genotype on susceptibility to *B. microti* showed that C3H mice reached higher peak parasitemia levels than BALB/C, C57BL6, CBA, and CF1 outbred mice, with the other mouse strains showing different degrees of susceptibility [29].

Advanced age is strongly associated with disease severity in human babesiosis [8,30,31,32]. Surprisingly, little is known about the mechanisms that underlie age-associated susceptibility to babesiosis. However, a study using a clinical isolate (RM/NS strain) of *B. microti* showed DBA/2 mice were highly susceptible to infection compared to C57BL6 and BALB/c mice. The study also showed that age-dependent susceptibility was evident for DBA/2 mice but not C57BL6 or BALB/c mice. More studies are clearly needed to determine the degree to which the increased susceptibility of individuals with increasing age to *B. microti* is impacted by host genetics as well as the immune and non-immune mechanisms that underlie the increased susceptibility with age.

Only one previous study has, to our knowledge, directly addressed whether mice that resolved primary infection with *B. microti* are resistant to secondary challenge. The previous study addressed secondary challenge of BALB/c mice with *B. microti* (Munich strain) [23]. Antibody depletion of CD4 T cells, CD8 T cells and neutralization of IFN-γ showed that CD4 T cells and IFN-γ, but not CD8 T cells, were important for protection against primary infection and secondary challenge with *B. microti*. In contrast, immune sera were not protective, suggesting either B cells were not important for protection or that immune sera did not include sufficient levels of anti-*Babesia* antibodies to confer protection. In contrast to our current study, IFN-γ-deficient mice in the BALB/c genetic background were unable to clear primary infection with the Munich strain and or resist secondary challenge if parasites were killed during primary infection with drug treatment followed by a secondary challenge with parasites. These differences may be due to the use of a different mouse background (BALB/c versus C57BL6) and a different strain of *B. microti*.

## 5. Conclusions

Our results indicate that primary infection with *B. microti* can result in robust protection against secondary challenge with parasites. However, it is possible that no single aspect of the immune system is solely responsible for adequate protection against primary or secondary challenge with *B. microti*. Our data along with other studies show that CD4 T cells make an important contribution to both primary infection and secondary challenges with *B. microti*. Our study also suggests that antibody might play an important role in protection against secondary challenge while playing a more limited role in the primary immune response. These data reopen a debate into the importance of B cells and antibody in the control of *B. microti* infection, especially given the susceptibility of individuals treated with Rituximib and other agents that impair B cell function to persistent babesiosis as well as babesiosis refractory to current therapies [12,13,33,34]. Although an important function of CD4 T cells is the production of IFN-γ, the absence of IFN-γR did not compromise protection against secondary challenge with the parasite in our model. This suggests that the importance of CD4 T cells in C57BL6 mice does not depend on their production of IFN-γ. Future studies are needed to evaluate the degree to which host genotype impacts the outcome of infection with *B. microti* as well as other parasite species such as *B. duncani, B. duncani*-like species and *B. divergens*. Future studies examining immune and non-immune mechanisms that underlie disease severity in response to *Babesia microti* and other *Babesia* species are needed to evaluate the potential impact of both host and parasite genetics on the pathophysiology and immune response during human babesiosis.

## Data Availability

The raw data are included in the manuscript as parasitemia is shown in figures for each individual mouse.
